# Role of exosome-mediated molecules SNORD91A and SLC40A1 in M2 macrophage polarization and prognosis of ESCC

**DOI:** 10.1007/s12672-023-00797-x

**Published:** 2023-09-23

**Authors:** Yang Xue, Zhengyan Cheng, Yida Liao, Xing Chen

**Affiliations:** 1 Department of Thoracic Surgery, Sichuan Academy of Medical Sciences & Sichuan Provincial People’s Hospital, Chengdu, China; 2 Department of Pathology, Sichuan Academy of Medical Science & Sichuan Provincial People’s Hospital, Chengdu, China

**Keywords:** ESCC, Exosomes, M2 Macrophage Polarization, SNORD91A, SLC40A1

## Abstract

**Background:**

Exosome-mediated interaction serves as a significant regulatory factor for M2 macrophage polarization in cancer.

**Methods:**

All accessible data were acquired from The Cancer Genome Atlas (TCGA) database and analyzed using R software. Molecules implicated in exocrine secretion were amassed from the ExoCarta database. Our research initially quantified the immune microenvironment in Esophageal Squamous Cell Carcinoma (ESCC) patients based on the expression profile sourced from the TCGA database. Additionally, we delved into the biological role of M2 macrophages in ESCC via Gene Set Enrichment Analysis (GSEA).

**Results:**

We observed that patients with high M2 macrophage infiltration typically have a poorer prognosis. Subsequently, a total of 1457 molecules were identified, with 103 of these molecules believed to function through exocrine mechanisms, as supported by data from the ExoCarta database. SNORD91A and SLC40A1 were ultimately pinpointed due to their correlation with patient prognosis. Moreover, we investigated their potential roles in ESCC, including biological enrichment, immune infiltration, and genomic instability analysis.

**Conclusions:**

Our study identified exosome-associated molecules, namely SNORD91A and SLC40A1, which notably impact ESCC prognosis and local M2 macrophage recruitment, thereby presenting potential therapeutic targets for ESCC.

## Background

Esophageal cancer (EC) remains the eleven most prevalent cause of cancer and ranks sixth for cancer-related mortality in the world, accounting for 6, 04, 100 new cases and 5, 44, 076 cancer-related deaths annually [1]. The incidence rates of EC show an alarming increase of over sixfold worldwide [2]. Despite advancements in diagnostic and multimodality therapy, the treatment of EC is still confronted with problems as resistance to drugs and local neoplasm recurrence frequently which leads to poor efficacy of treatment [3]. Thus, the average 5-year survival for EC patients is approximately 20%, and most of the patients die soon after diagnosis [4]. The accurate diagnosis and prognosis assessment of EC patients will make individual treatment possible and optimize therapy management. Therefore, the identification of early diagnostic and prognostic biomarkers is significant in improving the survival rate of EC patients.

Macrophages, highly heterogeneous phagocytic cells, are important for the person to defend against pathogens and maintain homeostasis [5]. After being activated by inflammatory stimuli, macrophages induce an anti-inflammatory pattern in response to stimuli by exhibiting functional and phenotypic features (e.g., M1-like phenotype) [6]. However, as a constitutive part of the tumor microenvironment (TME), tumor-associated macrophages (TAMs) are involved in promoting malignant progression and metastasis [7]. TAMs with an inhibitory M2-like phenotype may undermine anti-tumor immunity and mediate tumor immune escape by inhibiting cytotoxic function and the release of effector factors of immune cells [8–10]. Therefore, there is a positive correlation between the proportion of TAMs in TME and the poor prognosis of patients with solid malignancies [11].

Exosomes, surrounded by a lipid bilayer, are 30–150 nm diameter membrane vesicles, which are secreted by a variety of cells and contain a substantial of biomolecules, such as nucleic acids, proteins and enzymes [12, 13]. Exosomes play an important part in tumorigenesis, tumor progression, and metastasis [14]. By affecting gene expression levels and delivering genetic information to regulate cellular activities, exosomes can accelerate tumor growth, angiogenesis and weaken the response to immune checkpoint inhibitors (ICIs) and then lead to drug resistance [15–17]. Additionally, exosomes can promote immune invasion mediated by TAMs [18]. These characteristics enable exosomes as possible targets to enhance the sensitivity of immunotherapy of cancers. It is significant to identify prognostic signatures related to TAMs and exosomes in EC patients.

The rapid development of bioinformatics has provided convenience for researchers [19–22]. Here, we firstly quantified the immune microenvironment of esophageal squamous cell carcinoma (ESCC) patients based on the expression profile obtained from the TCGA database. We found that the patients with high M2 macrophage infiltration tend to have poor prognosis. Also, we explored the biological role of M2 macrophages in ESCC through the GSEA analysis. Next, a total of 1457 molecules were identified. Among these molecules, 103 molecules were regarded to exert their role in an exocrine manner based on the evidence from the ExoCarta database. SNORD91A and SLC40A1 were finally identified for their correlation with patients prognosis. Further, we explored their underlying role in ESCC, including biological enrichment, immune infiltration, and genomic instability analysis.

## Methods

### Public data collection

The transcription profile information and corresponding clinical parameters of each ESCC sample were obtained from The Cancer Genome Atlas (TCGA) database through the TCGA-GDC project (https://portal.gdc.cancer.gov/). Briefly, the original and individual gene expression files (STAR-Counts) files were downloaded and collated using the author R code. Samely, the initial clinical information was get in a “bcr-xml” form and collated using the author Perl code. The list of molecules involved in exocrine secretion was collected from the ExoCarta database (http://www.exocarta.org). All the data were pre-processed before analysis. The value of tumor mutational burden (TMB) and microsatellite instability (MSI) were obtained from the TCGA database.

### Immune microenvironment quantification

The quantification of the ESCC immune microenvironment was conducted using the CIBERSORT algorithm [23]. The quantified immune cell includes M2 macrophages.

### Pathway enrichment analysis

The potential biological differences between the two specific groups were investigated using the Gene set enrichment analysis (GSEA) [24]. The Hallmark and Gene Ontology (GO) and Kyoto Encyclopedia of Genes and Genomes (KEGG) gene sets were identified as the reference gene set. The GO analysis included biological process (BP).

### Identification of the prognosis-related molecules

For the input molecules, the univariate Cox regression analysis was utilized to identify the molecules tightly associated with the patients prognosis. Furthermore, LASSO regression was performed for data dimension reduction and optimization variable screening [25]. The LASSO regression, with its ability to select a more refined and meaningful set of predictive variables, played a crucial role in enhancing the robustness and interpretability of the model, thereby contributing significantly to the success of the study [26, 27]. Ultimately, the multivariate Cox regression analysis was performed to screen the prognosis-related genes.

### Single-sample GSEA (ssGSEA)

The up- or down-regulated effect of SLC40A1 and SNORD91A on specific immune cells was assessed using the ssGSEA algorithm [28].

### Statistical analysis

The analysis of public data was conducted using the R software. For the data that had a normal distribution, a Pearson correlation analysis was carried out. When analyzing data with a nonnormal distribution, the Spearman Rank test was utilized. The P value used was two-sided and considered statistically significant when less than 0.05.

## Results

### The role of M2 macrophages in ESCC

**Fig. 1:**
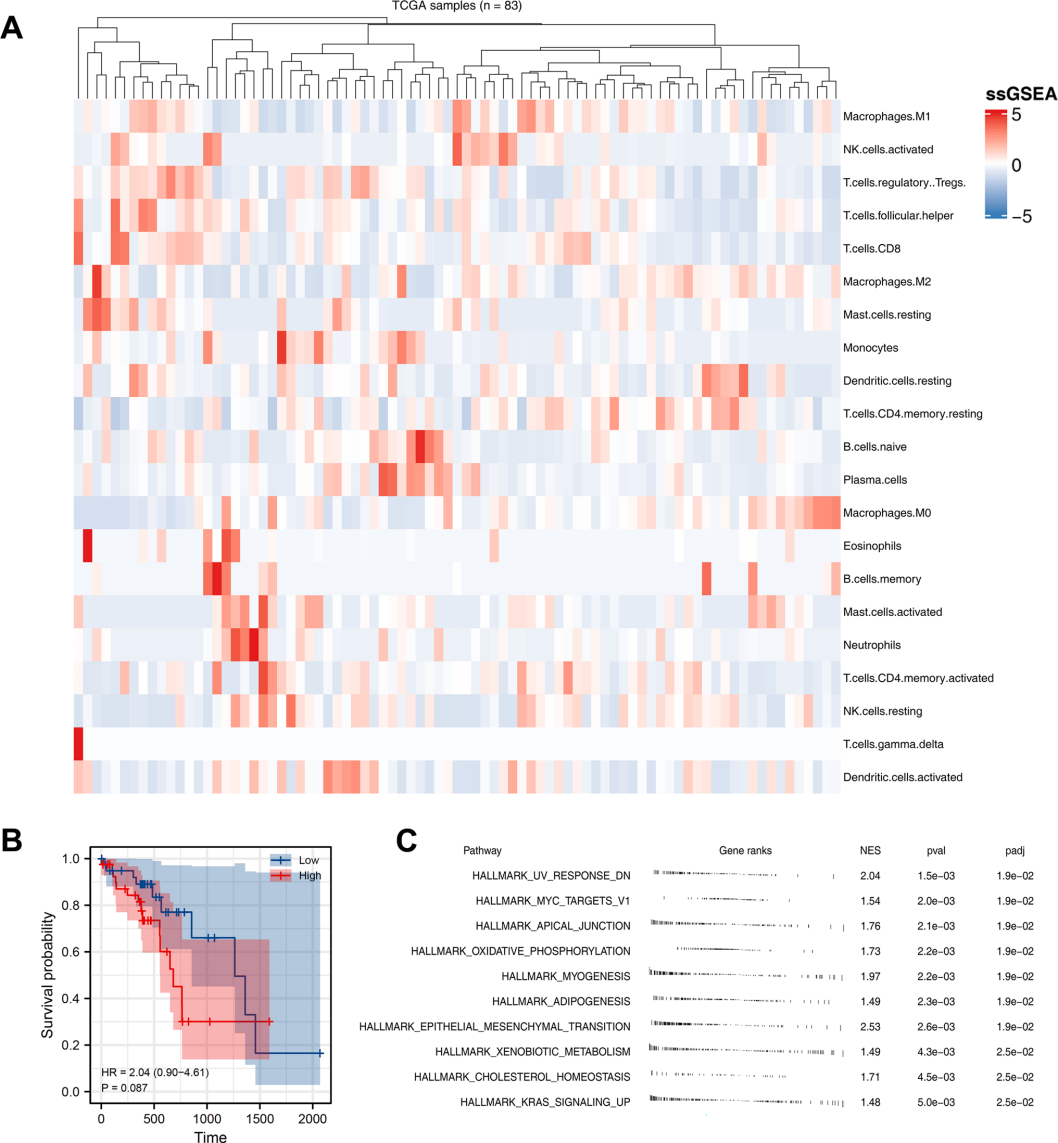
M2 macrophages in ESCC. **A**: the CIBERSORT algorithm was utilized to quantify ESCC immune microenvironment; **B**: KM survival curves of patients with high and low M2 macrophage infiltration; **C**: GSEA analysis of M2 macrophages

Firstly, based on the expression profile and CIBERSORT algorithm, we quantified the ESCC immune microenvironment, including M2 macrophages (Fig. 1A). Kaplan–Meier (KM) survival curves indicated that the patients with high M2 macrophage infiltration tend to have a worse prognosis (Fig. 1B, HR = 2.04, P = 0.087). Biological enrichment analysis showed that in patients with high M2 macrophages infiltration, the pathways of UV response DN, MYC targets, apical junction, oxidative phosphorylation, myogenesis were significantly activated (P = ?) (Fig. 1C).

### Identification of the exosomes-related molecules associated with M2 macrophage infiltration

**Fig. 2:**
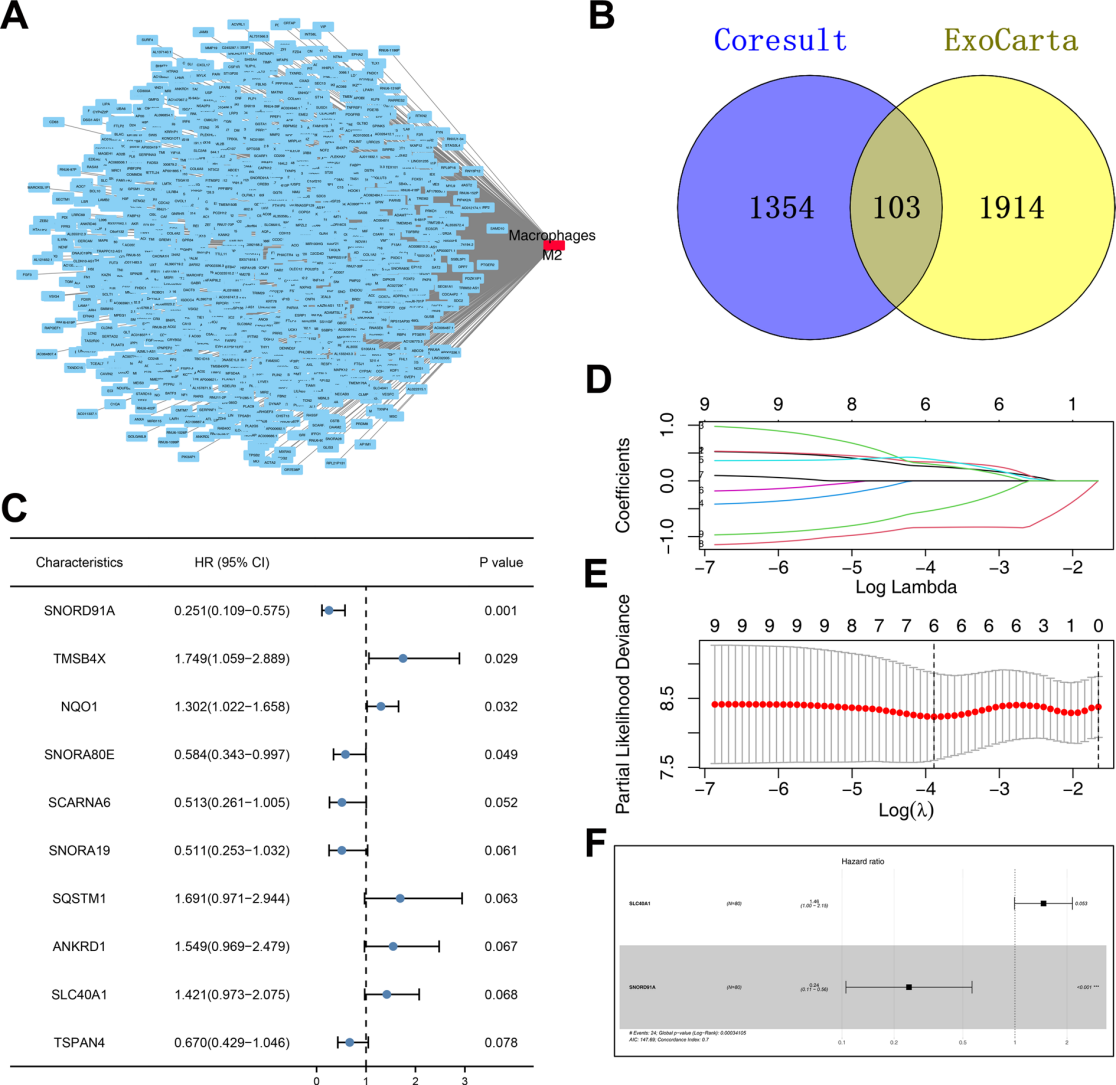
Identification of the exosomes-related molecules associated with M2 macrophage infiltration. **A**: a total of 1457 molecules were identified with a strong correlation with M2 macrophages; **B**: among the above 1457 molecules, 103 molecules were identified as exocrine-related molecules based on the evidence from the ExoCarta database; **C**: Univariate Cox regression analysis of 103 molecules; **D**–**E**: LASSO regression analysis; **F**: Multivariate Cox regression analysis

Next, we try to screen the molecules with a strong correlation with M2 macrophages (|Cor|> 0.3 and P < 0.05) and a total of 1457 molecules were identified (Fig. 2A). Among these molecules, 103 molecules were regarded to exert their role in an exocrine manner based on the evidence from the ExoCarta database (Fig. 2B). Considering the relatively small sample counts of TCGA-ESCC, we broaden the threshold of P to 0.1. Through the univariate Cox regression analysis, we found that the genes SNORD91A, TMSB4X, NQO1, SNORA80E, SCARNA6, SNORA19, SQSTM1, ANKRD1, SLC40A1 and TSPAN4 were remarkably correlated with patients prognosis (Fig. 2C). LASSO regression algorithm was used for data dimension reduction (Fig. 2D, E). Finally, multivariate Cox regression analysis identifies two exosome-related molecules SNORD91A and SLC40A1 (Fig. 2F).

### Prognosis analysis of SNORD91A and SLC40A1

**Fig. 3:**
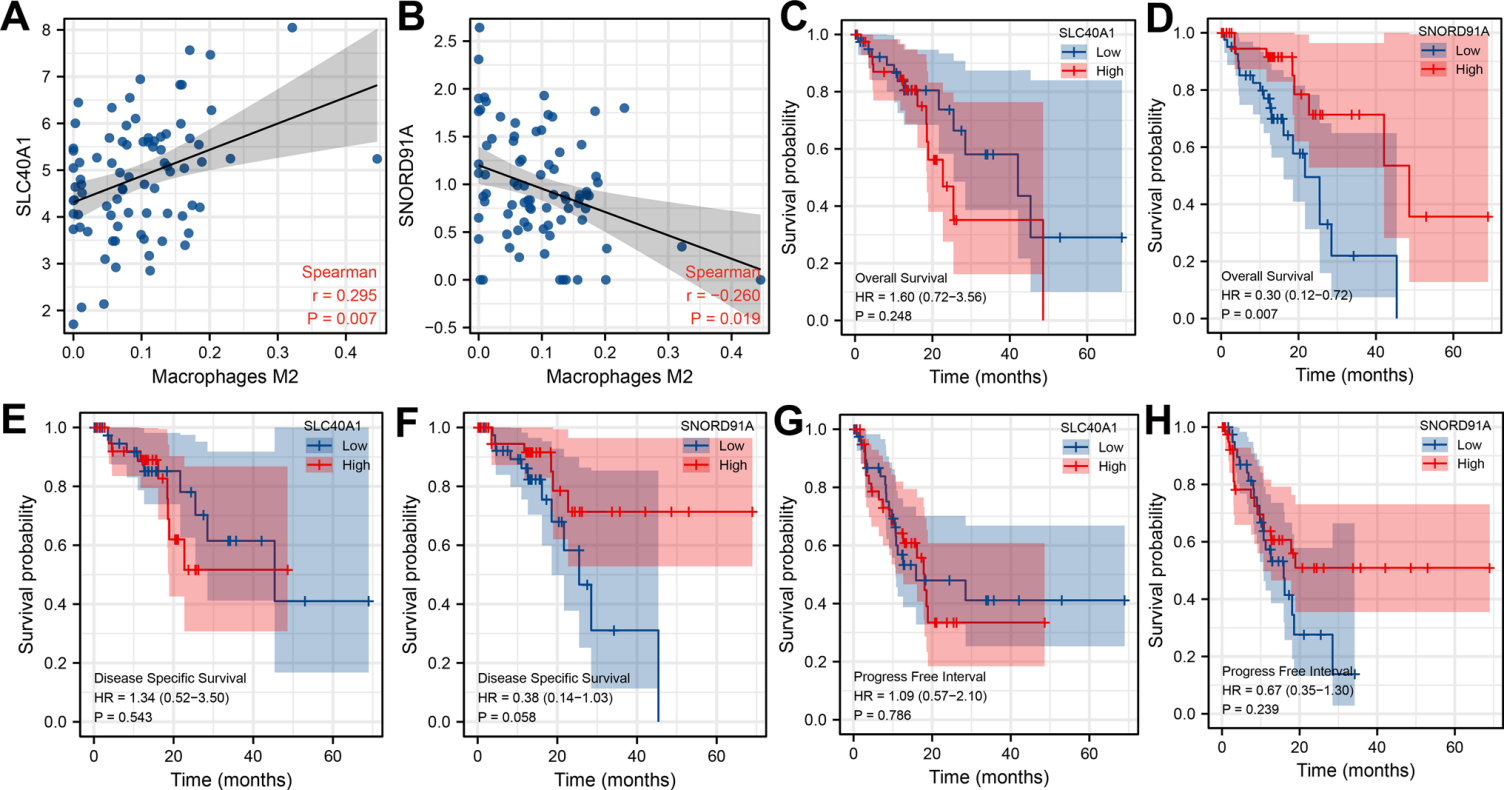
Prognosis analysis of SNORD91A and SLC40A1. **A**, **B**: correlation of SNORD91A and SLC40A1 with M2 macrophages; **C**, **D**: KM survival curves of OS (SNORD91A and SLC40A1); **E**, **F**: KM survival curves of DSS (SNORD91A and SLC40A1); **G**, **H**: KM survival curves of PFI (SNORD91A and SLC40A1)

Next, we found that the SLC40A1 was positively correlated, yet SNORD91A was negatively correlated with M2 macrophage infiltration (Fig. 3A, B). KM survival curves of overall survival indicated that SLC40A1 might be associated with poor prognosis, while SNORD91A was correlated with better prognosis (Fig. 3C, D). Although the P value is not significant, KM survival curves of disease-free survival (DSS) seems to show the same trend (Fig. 3E, F). However, no significant difference was observed in progression-free survival (PFI) (Fig. 3G, H).

### Biological enrichment of SNORD91A and SLC40A1

**Fig. 4:**
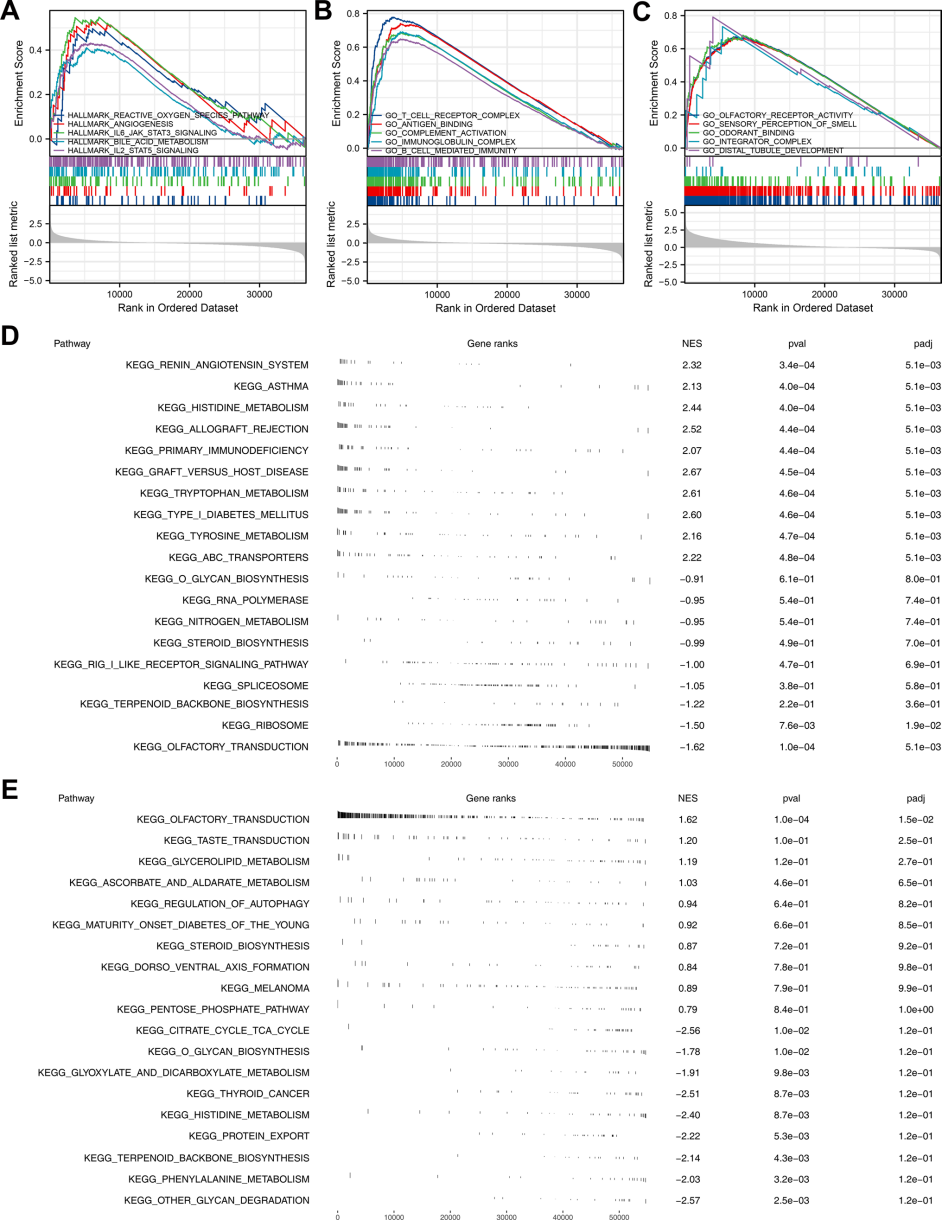
Biological enrichment. **A**: GSEA analysis of SLC40A1 based on Hallmark gene set; **B**: GSEA analysis of SLC40A1 based on GO gene set; **C**: GSEA analysis of SNORD91A based on Hallmark gene set; **D**: GSEA analysis of SLC40A1 based on KEGG gene set;** E**: GSEA analysis of SNORD91A based on KEGG gene set

Then, we try to investigate the underlying biological role of SNORD91A and SLC40A1 in ESCC patients. GSEA analysis based on the Hallmark gene set indicated that the terms of reactive oxygene species pathway, angiogenesis, IL6/JAK/STAT3 signaling, bile acid metabolism, IL2/STAT5 signaling were significantly activated in patients with high SLC40A1 expression (Fig. 4A). GSEA analysis based on the GO gene set showed that the pathway activity of T cell receptor complex, antigen binding, complement activation, immunoglobulin complex, B cell-mediated immunity were significantly upregulated in patients with high SLC40A1 level (Fig. 4B, C). Meanwhile, GSEA analysis based on the GO gene set indicated that in patients with high SNORD91A level, the terms of olfactory receptor activity, sensory perception of smell, odorant binding, integrator complex, distal tubule development were significantly activated (Fig. 4D). GSEA analysis based on the KEGG gene set showed that the terms of the renin-angiotensin system, asthma, histidine metabolism were significantly upregulated in patients with high SLC40A1 expression (Fig. 4E). GSEA analysis based on the KEGG gene set showed that the terms of olfactory transduction, taste transduction and glycerolipid metabolism were significantly upregulated in patients with high SNORD91A expression.

### Immune-related analysis of SNORD91A and SLC40A1

**Fig. 5:**
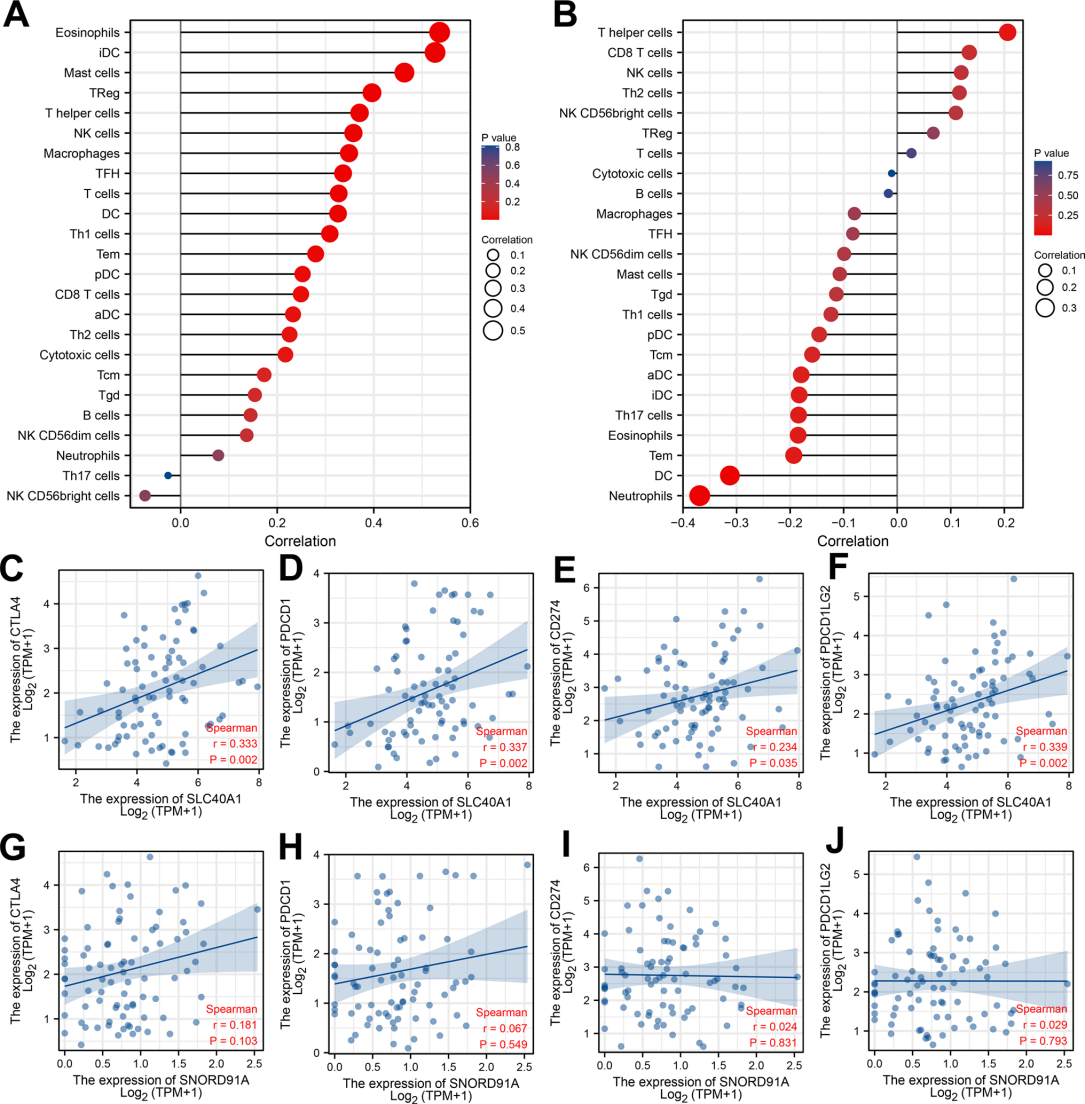
Immune-related analysis of SNORD91A and SLC40A1. **A**: immune infiltration analysis of SLC40A1 based on ssGSEA algorithm; **B**: immune infiltration analysis of SNORD91A based on ssGSEA algorithm; **C**–**F**: correlation of key immune checkpoints with SLC40A1; **G-J**: correlation of key immune checkpoints with SNORD91A

Immune infiltration analysis indicated that SLC40A1 was positively correlated with eosinophils, iDC, mast cells, TReg, T helper cells (Fig. 5A); SNORD91A was positively correlated with T helper cells, CD8 T cells and NK cell, while negatively correlated with neutrophils, DC and Tem (Fig. 5B). Furthermore, we evaluate the correlation between these two molecules and important immune checkpoints. Results indicated that SLC40A1 was positively correlated with CTLA4, PDCD1, PDCD1LG2 and CD274 (Fig. 5C–F). However, no remarkable correlation was found in SNORD91A (Fig. 5G–J).

### Genomic instability

**Fig. 6:**
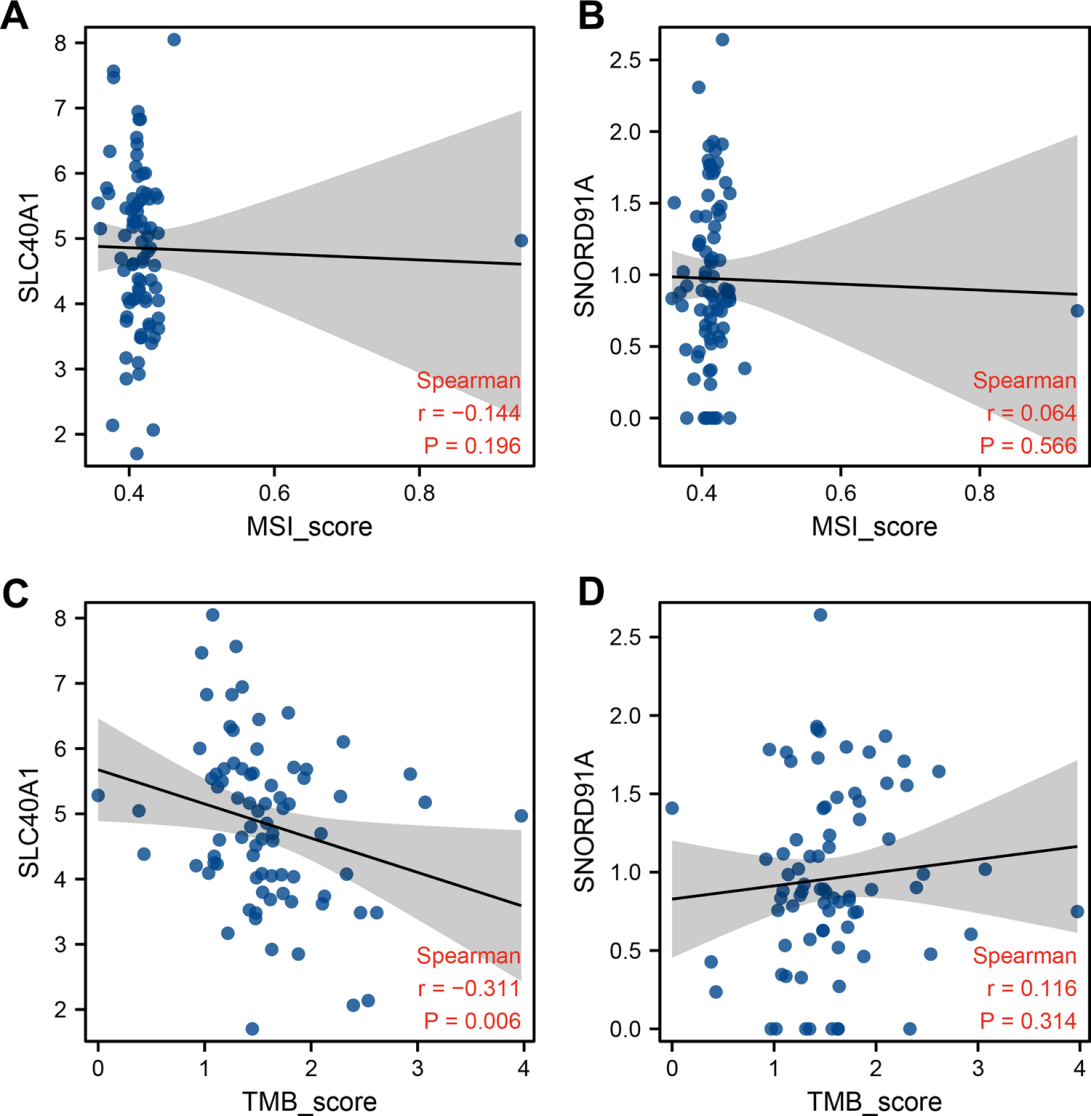
Genomic instability. **A**: correlation of MSI and SLC40A1; **B**: Correlation of MSI and SNORD91A; **C**: correlation of TMB and SLC40A1; **D**: correlation of TMB and SNORD91A

We next evaluated the effect of SNORD91A and SLC40A1 on genomic instability. The result indicated that SCL40A1 had no significant effect on TMB and MSI (Fig. 6A, B). Nonetheless, a negative correlation was found between SNORD91A and TMB score (Fig. 6C), but not MSI score (Fig. 6D).

## Discussion

EC remains an extremely threatening disease globally [29]. Despite rapid advancements in medical technology, EC often does not have an excellent prognosis. In general, EC presents with relatively hidden symptoms, has a poor prognosis and a high metastatic potential [30]. Consequently, the exploration of novel biomarkers of EC with the potential for clinical application is meaningful.

In our study, we firstly quantified the immune microenvironment of ESCC patients based on the expression profile obtained from the TCGA database. We found that the patients with high M2 macrophage infiltration tend to have poor prognosis, consistently with previous study conducted in lung squamous cell carcinoma [34]. Also, we explored the biological role of M2 macrophages in ESCC through the GSEA analysis. Next, a total of 1457 molecules were identified. Among these molecules, 103 molecules were regarded to exert their role in an exocrine manner based on the evidence from the ExoCarta database.

More importantly, SNORD91A and SLC40A1 were identified for their correlation with patient’s prognosis, e.g., M2 macrophages and exocrine secretion. In ovarian cancer, Wu et al. found that the SLC40A1 was associated with cisplatin resistance, which was mediated by miR-194-5p [31]. In prostate cancer, Liang et al. found that SLC40A1 can promote cancer cell proliferation and was regulated by miR-18a-5p [32]. In liver cancer, Hu et al. demonstrated that the interaction between HAMP from hepatocytes and SLC40A1 from macrophages can facilitate cancer cell proliferation [33]. In human glioblastoma, SLC40A1 was found to induce ferroptosis and affect cell viability [34]. There are few studies focused the role of SNORD91A in cancer at present. Our results provide novel insights into SNORD91A and SLC40A1 in ESCC and improved their functional network.

Meanwhile, GSEA analysis based on the Hallmark gene set indicated that reactive oxygen species pathway, angiogenesis, IL6/JAK/STAT3 signaling, bile acid metabolism, IL2/STAT5 signaling were significantly activated in patients with high SLC40A1 expression. EC is a disease with high metastatic potential. Also, angiogenesis is important for cancer distant metastasis [35]. Meng et al. revealed that acid/bile exposure can trigger TRAIL-mediated apoptosis in EC cells by suppressing the decoy receptors and c-FLIPR [36]. Shi et al. indicated that calreticulin can enhance EC migration and invasion by upregulating neuropilin-1 expression in a STAT5A-dependent manner [37]. Consistently, GO and KEGG analysis also indicated that SNORD91A and SLC40A1 might exert their role through the enriched biological pathways.

Immune infiltration analysis indicated that SLC40A1 was positively correlated with eosinophils, iDC, mast cells, TReg, T helper cells; SNORD91A was positively correlated with T helper cells, CD8 T cells and NK cell, while negatively correlated with neutrophils, DC and Tem. The complex immune microenvironment in and around tumor can affect tumor progression [38]. Our results showed that SNORD91A and SLC40A1 can induce local local immune microenvironment remodeling, further affecting tumor development.

Even though we used reliable public data and conducted high-quality analysis, we still need to point out some limitations. Firstly, it is undeniable that the number of ESCC samples obtained is relatively small (less than 100). Therefore, sample bias is unavoidable. We will be able to draw more reliable conclusions after including more ESCC data in the future. Secondly, SNORD91A and SLC40A1 have been identified as exosome-related molecules through the ExoCarta database. However, further experimental validation of SNORD91A and SLC40A1 in ESCC has not been completed.

## Conclusion

The SNORD91A and SLC40A1 as the exosome-associated molecules could impact the prognosis of ESCC and the recruitment of local M2 macrophage, possibly serving as the therapeutic targets for ESCC. However, the small sample size analysed limited the reliability of findings, requiring a further analysis of a bigger database.

## Data Availability

The original contributions presented in the study are included in the article/supplementary material, further inquiries can be directed to the corresponding author.
